# Factors Influencing British Adolescents’ Intake of Whole Grains: A Pilot Feasibility Study Using SenseCam Assisted Interviews

**DOI:** 10.3390/nu11112620

**Published:** 2019-11-01

**Authors:** Maya Kamar, Charlotte Evans, Siobhan Hugh-Jones

**Affiliations:** 1 Nutritional Epidemiology Group (NEG), School of Food Science and Nutrition, University of Leeds, Leeds LS2 9JT, UK; 2 School of Psychology, Faculty of Medicine and Health, University of Leeds, Leeds LS2 9JT, UK; s.hugh-jones@leeds.ac.uk

**Keywords:** whole grain, fibre, adolescents, SenseCam, interviews

## Abstract

High whole grain intake is beneficial for health. However, adolescents consume low levels of whole grain and the understanding of the underpinning reasons for this is poor. Using a visual, participatory method, we carried out a pilot feasibility study to elicit in-depth accounts of young people’s whole grain consumption that were sensitive to their dietary, familial and social context. Furthermore, we explored barriers and suggested facilitators to whole grain intake and assessed the feasibility of using SenseCam to engage adolescents in research. Eight British adolescents (aged 11 to 16 years) wore a SenseCam device which auto-captured images every twenty seconds for three consecutive days. Participants then completed traditional 24-hour dietary recalls followed by in-depth interviews based on day three SenseCam images. Interview data were subjected to thematic analysis. Findings revealed that low adolescent whole grain intake was often due to difficulty in identifying whole grain products and their health benefits; and because of poor availability in and outside of the home. The images also captured the influence of parents and online media on adolescent daily life and choices. Low motivation to consume whole grains, a common explanation for poor diet quality, was rarely mentioned. Participants proposed that adolescent whole grain consumption could be increased by raising awareness through online media, improved sensory appeal, increased availability and variety, and tailoring of products for young people. SenseCam was effective in engaging young people in dietary research and capturing data relevant to dietary choices, which is useful for future research.

## 1. Introduction

Whole grains are a source of dietary fibre and are rich in protein, vitamins, minerals, and phyto-chemicals [1,2,3]. Systematic reviews indicate that high whole grain consumption may lead to improved insulin sensitivity and reductions in blood pressure, total and low density lipoprotein (LDL) cholesterol, colorectal cancer, breast cancer, cardiovascular disease (CVD) risk [4,5,6,7,8,9,10,11], although the evidence on improved weight status and reduced waist circumference is less consistent [12]. It has been suggested that a daily intake of around one to three 30 g servings of whole grain foods substantially reduces the risk of disease outcomes [3,13,14]. Although the U.S. Department of Agriculture (USDA) recommends three or more ounce-equivalents/day of whole grain for adults and 1.5 to 4 ounce-equivalents/day for children/adolescents [15], national data show that the mean intake among American adults and children/adolescents is much lower, at around 0.82 and 0.57 ounce-equivalents/day, respectively [16]. Similarly low levels of intake are reported in the United Kingdom (UK). The UK’s National Dietary Survey of British Adults (NDNS) (2008–2011) reported that 18% of adults and 15% of children/adolescents do not consume any whole grain foods, with the median intake for adults and children/adolescents being around 20 g/day and 13 g/day, respectively [17,18]. In the UK, adolescents and individuals from lower socio-economic groups appear to have the lowest levels of intake [17,18,19].

Most studies exploring whole grain intake correlates have been conducted on other age groups or on non-UK adolescents [20,21]. Previous research has reported the following as possible barriers to whole grain intake: lack of awareness and misconceptions about whole grain food products; inability to identify them; lack of awareness of the health benefits; perceived or experienced negative sensory properties; high price; low availability and accessibility; and lack of knowledge of preparation techniques [22]. National studies clearly reveal the need to target UK adolescents to improve their whole grain intake. Doing so could have significant benefits to health in the short- and long-term and potentially the health of their own families in the future [23,24,25].

**Figure 1:**
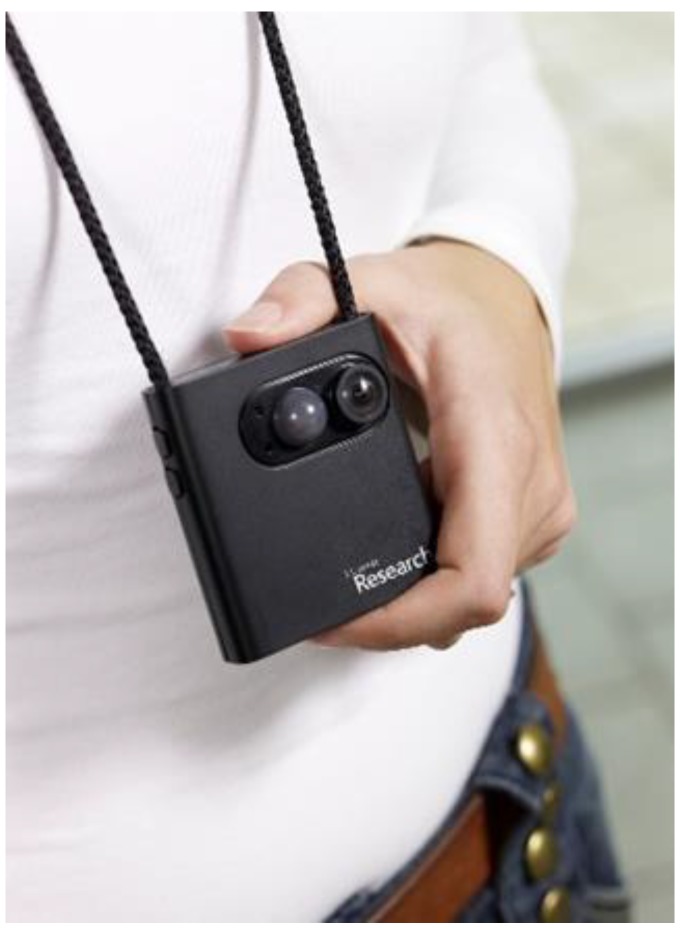
The Microsoft^®^ SenseCam digital camera and how it is worn by participants.

There are challenges when researching factors influencing adolescent eating habits. Focus groups with adolescents have revealed a strong effect of peer influence but may have led to a restricted expression of views based on the group as a whole rather than views of individuals. In addition, the difficulties associated with whole grain identification has prompted a need for in-depth exploration of the topic [22]. Furthermore, traditional methods of surveys or interviews have been critiqued as inadequate for capturing the complexity of factors influencing dietary behaviour [26]. Adolescents’ dietary behaviour is shaped by everyday contexts, such as family and school. We need to develop methods to engage young people in dietary research and to capture the key contextual influences on their dietary behaviour to better understand barriers to consumption and potential opportunities for intervention. Visual methods of data collection and analysis are gaining popularity in health research [27], and are advocated by The Lancet Commission on adolescent health to increase engagement and participant-led research and the potential for new insights. This study aimed to explore the feasibility of young people using a camera called SenseCam (developed by Microsoft® Research, Cambridge, UK; see Figure 1) to enhance the exploratory interviews. SenseCam is a wearable camera which hangs from the neck and auto-captures approximately 3600 first-person point-of-view digital images per day.

This technology was initially used in research with memory-impaired patients to capture and aid in recalling details of daily life [28]. SenseCam has since been used in a variety of health research projects, including physical activity and nutrition mainly with adults [29,30,31,32]. A few SenseCam studies have involved adolescents, some of which included documenting and measuring active and sedentary behavior [33], as well as measuring built environmental features that impact physical activity [34]. These studies offered quantitative analysis of the SenseCam images and feasibility testing of the technology. One recent study used focus groups to understand adolescents’ experiences of using SenseCam, in a study measuring daily exposure to food marketing across media to explore determinants of health [35]. However, to date, no studies have explored the potential of SenseCam to help us understand influences on adolescent whole grain intake. SenseCam images can be used to scaffold interviews, helping people to remember dietary choices, and to provide and explain context. This could generate novel insights into the real-world dietary behaviour of adolescents. Our pilot feasibility study aimed to generate new insights into whole grain intake in ways that are sensitive to lived experience and context, explore barriers and suggested facilitators to whole grain intake and assess the feasibility of using SenseCam to engage and work with adolescents.

## 2. Methods

### 2.1. Ethics and Participant Recruitment

The University of Leeds MEEC Faculty Research Ethics Committee approved the study protocol (MEEC 13-015, date of approval 9 April 2014). This study adhered to the guidelines laid down in the Declaration of Helsinki. Head teachers and all adolescent participants provided written informed consent, along with parental/carer assent. Guidelines and recommendations on ethical use of SenseCam were utilised from previous studies [36].

Participants were recruited via school contacts and word of mouth. Participants interested in taking part were given an overview of the study and information sheets, and were invited to email or telephone the researcher if they were interested in participating. The maximum number for recruitment was ten adolescents due to the availability of one SenseCam device. Moreover, saturation would be expected to be reached by this number based on similar research in other areas [28,29,30,32,33,34,35]. Therefore, recruitment was stopped after ten participants expressed interest in the project.

### 2.2. Using SenseCam

The study was single-blinded, in that participants were told that the researcher was interested in adolescent lifestyle and the factors that influence this. Interest in dietary intake and the focus on whole grain was not revealed to the participants in order to limit bias and the possibility of altered behaviour.

The device used in this study was the Microsoft^®^ SenseCam, which auto-captured images every 20–30 s. One device was available throughout the study, and participants used it in turn. After signing the consent forms at the first meeting with the researcher, the participants were briefed on the study and SenseCam. They used the SenseCam for three days, followed by an interview on day four. Participants were advised that they could use the pause button on the SenseCam device, which freezes image auto-capture for five minutes. They were allowed to remove it in situations of discomfort or locations where objection or unwanted attention would occur, such as in private gatherings or places of worship. Participants were requested to ask permission to use SenseCam at school and were encouraged to explain the purpose of the camera if asked. They were provided with a script with details of the research and data confidentiality to use in these situations.

### 2.3. In-Depth Interviews

At interview start, traditional 24-hour dietary recalls of day three were conducted, with the aid of the Food Standard Agency’s (FSA) Photographic Atlas of Food Portion Sizes [37,38]. Results of the 24-hour recalls are not reported here. Following the 24-hour recalls, the SenseCam images were uploaded to a secure, password-protected study file. In line with our ethical protocol, participants were given time to privately check and remove any of the uploaded SenseCam images before proceeding to the interview.

Interviews were semi-structured (see Supplementary Materials (Table S1) for the interview schedule), and directly assisted by the participant approved SenseCam images from day three. Day three was chosen as participants’ memory of the day was likely to be more reliable and participant behavior more natural as they adjusted to SenseCam wear over the three days. Approximately 1900 day three images were available per participant for use in the interview. The interview began by focusing on broad questions about adolescent lifestyle. Early in the interview, the true focus of the research was revealed and the interview focused in on dietary choice. SenseCam images were displayed on a computer screen and participants scrolled through images and either chose to stop at a particular image or were asked to stop at one by the researcher (often related to meals or discussed topics). The interviewer encouraged participants to express their opinions freely and used open, non-leading questions and interviews oscillated between researcher-led and participant-led questions and comments. At a later point in the interview, and in order to encourage participants to express opinions on how to increase whole grain intake, information on the definition and health benefits of whole grains was provided. Upon completion of the interviews, participants were provided with vouchers and a certificate to thank them for their contribution to the research project. Interviews were audio-recorded and lasted approximately 75 min each.

### 2.4. Data Analysis

The interview recordings were transcribed by the first author, with all identifying information removed. Data were analysed using inductive thematic analysis as described by Braun and Clarke [39]. NVivo software was used to aid in management of data analysis (NVivo qualitative data analysis Software, 2012). First, transcripts were read carefully line by line and assigned descriptive labels. Second, units of text containing common labels were assigned provisional codes. Interviews and coding continued until no new codes were generated (data saturation). This was reached after eight interviews. At this point, codes (linked to the original text) were screened and those relevant to the research question were grouped into common themes. Emergent themes were discussed with the second and third authors and credibility checks were conducted (i.e., that the interpretation of the data were credible for their assignment to a theme and that there was sufficient evidence to support a theme). The third and final stage of analysis involved a review and refinement of the themes for ease of data presentation.

## 3. Results

Ten participants were recruited. Two participants dropped out due to family concern over SenseCam use with regards to privacy issues and the possibility of negative attention. The final sample of eight participants were aged 11 to 16 years old (median age: 13.5 years; see Table 1). Participants were British adolescents with a mixture of ethnic backgrounds, and there were equal numbers of males and females. Only three out of eight participants were given permission to use SenseCam at school, and the remaining participants used it outside school hours and during the weekend.

### 3.1. Factors Influencing Whole Grain Consumption

This sample of young people appeared relatively interested in health. Five out of eight participants spontaneously reported that they tried to eat healthily although they felt this was hard to do in practice. Their motivation to eat well was driven by looking and feeling good, weight management and longer-term health. Factors influencing whole grain consumption have been categorised under five themes: confusion and uncertainty, taste, home availability and influence, availability and accessibility beyond home, peer and social norms.

#### 3.1.1. Confusion and Uncertainty

**Figure 2:**
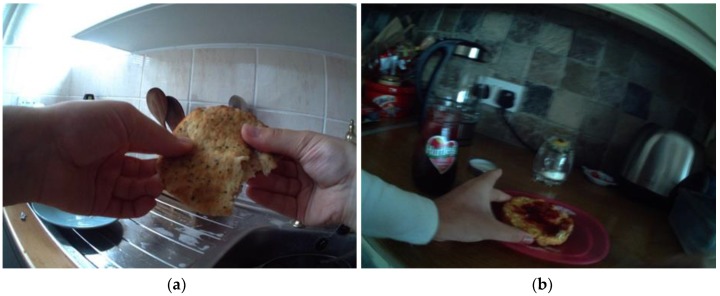
SenseCam images highlighting confusion in whole grain identification (Sasha and Emma) including seeded white bread (**a**) and rice cakes (**b**).

When asked about whole grain foods, most participants (by “most” we mean six or seven participants out of eight) perceived them as a “*healthier version of something [they] already ate*” (Sasha, F., 12 years). However, most participants were unsure about how to identify a whole grain product, and were confused about what made it a whole grain food and why it had health benefits. Two images of participants choosing food revealed this confusion. First, in reference to Figure 2a, the participant described seeded white bread as wholemeal toast, and second, in reference to Figure 2b, the participant thought her rice cake (and all rice cakes) were whole grain by default.

Five participants thought that colour was the main identification marker of whole grain products, and seven thought of wholemeal bread as the most obvious form of whole grain food.

*“I try to go for the brown-looking varieties, as I’ve heard that brown bread is healthier, but that’s it. I don’t know why it is healthier and what’s healthy about it*” (Liam, M., 16 years).

There was an assumption that whole grain was bread with added seeds or made with organic wheat which increased its fibre content. Another participant thought that whole grain products must contain less sugar, which was why it was healthier and recommended for people with diabetes, unlike “white bread”. Another participant mentioned that his father ate “*those healthy breads with fibre which filled you up right away”* (Peter, M, 13 years). Participants who mentioned fibre (five out of eight) stated it was better for digestion and *“helped food travel in the intestines*”, but were generally unsure of this. Only one participant said they might examine the product ingredient label to assess its whole grain status (without knowing the research was on whole grains): “*here I was reading the labels. It would usually say whole grain somewhere on the front. Because if it was whole grain then the company is like (sic) proud and literally wants everyone to know*” (Peter, M., 13 years).

Overall, participants were confused about what constituted a whole grain product, why it was healthy and how to identify whole grain foods. At this point in the interview, all participants were taught by the interviewee how to identify a whole grain product.

#### 3.1.2. Taste

Although participants expressed various opinions about whole grain foods, they all stated that the texture was dry—mainly as they had wholemeal bread in mind. Negative perceptions of texture were often combined with negative taste perceptions, and participants felt that these would need to be overcome to improve consumption: “*I would only think of eating whole grain one day in the future if I wanted to be healthy. But I don’t see myself liking it any time soon*” (Nathan, M., 13 years). One participant said that mixed varieties, such as 50-50 breads, were more acceptable in terms of taste and texture. Three participants expressed a preference for wholemeal vs. white bread for toasting, a preference stemming from habit through home availability (see Theme 3): It was a “*habit that became personal preference really*” (Olivia, F., 14 years). Some participants cited their preference for wholemeal bread as it was tastier, more “special”, filling, and healthier as white bread could “*make [her] fat*” (Hannah, F., 15 years). Following clarification of other whole grain foods, participants expressed favourable attitudes towards products such as wholemeal rolls, wraps, chapattis and rotis, and whole grain breakfast cereals. Most participants were pleasantly surprised to learn that other products such as bulgur wheat, brown rice, brown pasta, quinoa and popcorn were whole grain foods and expressed positive attitudes towards these based on taste and health benefits: “*Oh I love bulgur wheat, it’s so good! It has a really nice consistency because it’s slightly chewy but crunchy and nutty. It’s nice!*” (Olivia, F., 14 years); “*Ummm I’ve actually had some of it (whole grain pasta). I really like it because now I know it’s healthy and it still tastes nice at the same time!*” (Peter, M., 13 years). Thus, negative perceptions of whole grains were driven largely by the taste and texture of wholemeal bread, but more positive attitudes emerged when a broader range of foods were considered.

#### 3.1.3. Home Availability and Influence

SenseCam images indicated that home was still the main source of food for these young people, including food to be taken to school. The high number of SenseCam images capturing family meals led to discussions about the influence of the home environment and availability of whole grains on young people’s consumption. Reported home availability of whole grains ranged from a constant supply to little availability. One participant said, “*There is always a brown loaf in the house. There’s more often brown bread than white bread*” (Olivia, F., 14 years). An image of her mother cutting wholemeal bread, prompted the participant to explain how increased availability made her more likely to consume this type of whole grain food. Good availability of whole grain was also linked to parents of participants being described as health-conscious and who regularly provided whole grain foods at mealtimes. However, in the case of a few participants (by “few” we mean one or two participants), one or both parents preferred lower fibre varieties and this was cited as the reason for low availability in the home. Additionally, parental concern about food waste was perceived as a further factor that reduced whole grain purchases and availability.

**Figure 3:**
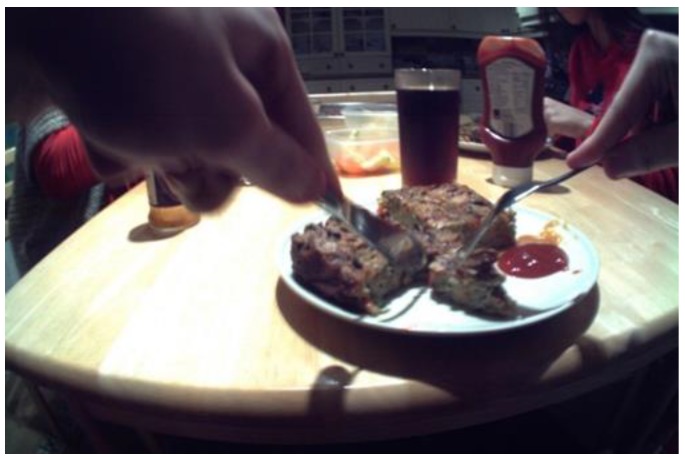
An example of cultural whole grain varieties consumed by participants at home: a bulgur-based omelette.

Cultural factors also influenced whole grain consumption as ethnic whole grain options were accepted and enjoyed by some participants (by “some” we mean three to five participants). Examples included rotis and chapattis, consumed by participants having South Asian origins, bulgur wheat by those having Turkish origins, and teff by those having African origins. Cultural varieties were described as fundamental to many family meals and were often the sole source of whole grain. While viewing the image of a homemade bulgar-based omelette (Figure 3), the interviewer asked the participant to explain the food shown in the image. This led to the discussion that he enjoyed this type of food, and that he had not been aware it was whole grain.

*“I’d have a chapatti or a roti with my dinner—I like those. But I wouldn’t go for the whole grain option otherwise like, say, in a sandwich or to school. I prefer white bread*” (Nathan, M., 13 years).

The home environment was influential not just through availability but also, for some, through parental modelling and expectations. Although two participants were sceptical of parental “advice”, e.g., “*my mum would tell me something because theoretically it is the “right” thing to do or heard it from culture.*” (Hannah, F., 15 years), most participants rated their family as their number one trusted source of health and dietary advice. The behaviour and beliefs of parents appeared influential to the dietary intake of the young participants. For example, in relation to consumption of fruit and vegetables, one participant stated: “*I guess it all eventually sinks in and becomes your own priority too.*” (Olivia, F., 14 years).

#### 3.1.4. Availability and Accessibility beyond Home

SenseCam images capturing food shopping prompted discussion of the availability and accessibility of whole grain foods in public spaces. Five participants reported that it was “*cheaper and easier to get white bread*” (Dylan, M., 11 years) than whole grain varieties and that this was a key factor limiting the participants’ ability to consume the recommended three portions per day. However, they felt that, compared to refined products, whole grain products were not as visible or “*out there*”, and that “*whole grain [varieties] would be somewhere at the top of the shelf or something, where you don’t notice them as much*” (Liam, M., 16 years). An image of supermarket shelves showing a dominance of white products lead the participants to reflect on this: “*If you go to the big supermarkets, you won’t see anything of that sort of stuff. You’d see the small stuff that are cultural, like a few pittas, maybe some roti. The bread section is just like being one whole shelf of white bread and then maybe, less visible, a few loaves of brown bread*” (Dylan, M., 11 years).

Most participants stated that it was difficult to access whole grain products in public. “*When you’re eating out I don’t think it’s available enough at all! Because when you see things like fast food or just general restaurants, if they do any kind of bread it’s always white bread.*” (Emma, F., 14 years). Another participant explained that, when eating out, “*You have to ask them to bring whole grain bread. And only a few places might have it*” (Hannah, F., 15 years). Accessing whole grain snacks in public spaces was seen as very difficult, as most vending machines in schools, hospitals, and public places “*never have whole grain cereal bars or the like*” (Nathan, M., 13 years). When asked about the availability of whole grain varieties in school, all participants stated that it was very hard or impossible to find them: “*school food is always pre-packed stuff, then they’re just ovened or microwaved. You would find croissants and, say, toast with butter. So it’s not usually proper food or even freshly cooked.*” (Peter, M., 13 years). Whole grain snack options (including cereal bars) were perceived to be limited in number and overpriced.

Discussions on the cost of food, and its influence on choice, was mixed. Most participants perceived white bread as cheaper, based on high demand and market competition, and whole grain foods as more expensive, but were unsure what processing methods were increasing the cost. For a few participants, item price was the dominant determinant of food choice outside the home, followed (according to one of the participant) by brand, sugar content and additives: “*people want the tastiest and the cheapest*” (Hannah, F., 15 years).

#### 3.1.5. Peer and Social Norms

Although the participants claimed that peers rarely directly influenced their food choice, some influence was reported following probing. According to one of the participants, in the back of one’s mind, there might be a fear of behaving outside of norms: “*They might start asking what is this stuff you’re eating there? And just the fact that you might be questioned or the slightest possibility of teased or mocked, especially by the boys, makes you think twice before doing anything that is remotely different than others*” (Olivia, F., 14 years). This also included consumption of more ethnic food types, as two of the participants pointed out. A participant stopped at the image of a takeaway outlet where he was buying a meal with his friends: “*You need to be the same as everyone else. Everything and anything that is different might be mocked*” (Nathan, M., 13 years). Participants reported that pressure to conform to norms dissipated somewhat during later adolescence. One participant explained changes during the last two years of secondary education: “*You start embracing the things you were taught and your own beliefs and hang around people who think similarly*” (Liam, M., 16 years). This participant reported his friends’ support (manifesting as lack of jeering) when ordering a salad or asking for the whole grain option.

**Figure 4:**
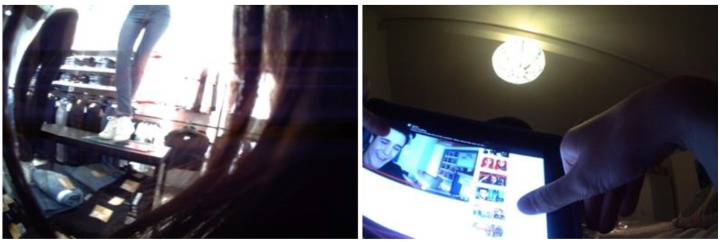
Clothes shopping and social media images prompted discussions around body image and the negative perceptions of carbohydrates.

SenseCam images of social media (e.g., Instagram photos) as well as clothes shopping prompted discussions about social norms for body shape (Figure 4). Carbohydrates were talked about in a negative way by participants of both genders, and participants felt that whole grains were carbohydrates they are meant to avoid. One young participant had engaged in carbohydrate-free dieting before deciding to manage her weight in a healthy way with an emphasis on whole grain: “*I eat whole grain when I am in diet-mode. It keeps me full and helps me lose weight. I read it online.*” (Sasha, F., 12 years). Several participants spoke of similar “days of feeling healthy”, where whole grains featured more predominantly, but it was nonetheless perceived as optional.

### 3.2. Improving Adolescent Whole Grain Consumption

Towards the final part of the interview, participants were asked to imagine that they were whole grain teenage ambassadors with the power and budget to intervene at any level to promote whole grain consumption among adolescents. Their suggestions spanned three themes.

#### 3.2.1. Promote Knowledge and Awareness

Participants believed that young people would consume more whole grain foods if they were more knowledgeable of their health benefits and were more aware of their nature: “*So that they actually know that whole grain is much better for you even though it may be more expensive or less ‘out there’... I don’t think most of them know about HOW much healthier it may be. And I think that would make them try to eat more whole grain*” (Peter, M., 13 years). They felt there should be more awareness of other whole grain products other than wholemeal bread, such as brown rice, brown pasta, wholemeal wraps, bulgur wheat and quinoa. Participants recommended persuasive media (spanning radio, TV, and online) to promote knowledge, including celebrity endorsement (although the credibility of this might be questioned by some): “*You have to do something really catchy to get people to see it or care. It has to be catchy enough or funny to be talked about or shared with friends so people would remember it.*” (Nathan, M., 13 years). Others suggested paying famous YouTubers with high numbers of young followers to promote whole grains as part of their healthy food blogging. According to the participants, this would spark interest and discussion: “*I watch a lot of YouTubers. they’re all eating more healthily and it’s like—quinoa! Wait, what is quinoa? Is that actually a healthy thing? Then I go ask my mum and look it up online and find out all about it.*” (Olivia, F., 14 years). One participant suggested that whole grain companies should sponsor sports events: “*If McDonald’s [is doing] it, then Kellogg’s certainly can!*” (Liam, M., 16 years).

Targeted campaigns to promote knowledge and awareness were advocated for some settings, for example, using public spaces where people were “*in the mood for being healthy*”, such as gyms and hospitals. However, prompting intake via school-based activities presented mixed views. Simply trying to promote whole grain intake in the form of posters or leaflets was perceived as ineffective, as students will “*look at it and just scoff or make fun of it*” (Hannah, F., 15 years). However, educating young people about whole grains was seen to be more promising, although experiences to date were poor. Most participants reported that they had, at some point, probably heard that “*Brown bread was better than any other bread—that’s what they said in school*” (Sasha, F., 12 years) but felt that there was inadequate coverage and discussion of how to identify whole grain products and understand their health benefits. Participants also complained that such sessions were too general, lecture-like, repetitive, and did not involve enough activities to provoke their interest or make the content memorable. However, school-based education which debunked myths about carbohydrates (and the dangers of cutting them) was reported as necessary and likely to be effective. “*Some teenagers think that healthier means almost no food, or no carbohydrates. Tell them to eat the right carbohydrates, not to eliminate them!*” (Hannah, F., 15 years). This was suggested as part of more engaging and informative education: “*I think a whole session in class should tackle this whole grain issue. It makes more sense in every single way: less processing, healthier, more environmentally friendly. It is convincing in every way, and it would lead to lots of discussions on how industry makes something less healthy the norm and people just follow through. These things don’t get discussed in class and I feel they should. I hadn’t even heard half the things I learnt about fibre today in school!*” (Sasha, F., 12 years).

#### 3.2.2. Changing Norms

Although increased knowledge and awareness was felt to be important, five out of eight participants stressed that the current perception of whole grains as “*special foods for extra health-conscious individuals*” (Liam, M., 16 years) limited consumption by young people. Participants emphasised the importance of shifting norms instead: “*Make it seem like a normal thing, rather than a special thing like only for healthy people... Make it dominate the market. Create varieties too... Get parents to give it to children when they’re little. Like white bread should get the special ‘white bread’ label and not whole grain, because whole grain is the norm of bread. Just like that*” (Olivia, F., 14 years). Improving availability was described as an important way to change norms, particularly in terms of removing less healthy choices. One participant argued that this is more important than knowledge in improving consumer choice: “*I think if I had that kind of budget and that kind of power I’d sort of force shops to reduce stocks of white bread, increase stock of brown bread and make that more often on the shelf and more obvious than white bread. I want white bread to be a lot rarer in shops. I don’t care if people don’t know what brown bread is and the benefits of it, I just want it to be available. It sort of makes it the norm.*” (Dylan, M., 11 years). Changing what is provided in schools was also recommended: “*I think the easiest way would be to get them to change the restaurant venues around the school which students flood out for lunch for to have brown bread. Oh and change the canteen!*” (Emma, F., 14 years). Shaping norms through changed availability could also be managed through cost. Participants suggested that white bread should be made more expensive than whole grain, and the money that is made through sales of white bread would offset the extra cost of increased whole grain production.

#### 3.2.3. Improving Appeal

Making whole grain products more appealing in their packaging was suggested as a key mechanism to promote the chances of young people choosing it. Currently, whole grain products are perceived as serious and boring, for people with special interests or who are fussy eaters, and are associated with “free-from” products. Whole grain products, they suggested, needed re-branding as normal and appealing for the average consumer: “*We want flashy colours, big fonts, and loads of colour. Why does the whole grain cereal look so much more dull and serious than a chocolate cereal?*” (Liam, M., 16 years). Packaging should also be improved in terms of clear labelling: “*Why should it be such a riddle to figure it out? There should be a large clear stamp, like a government-regulated thing, that says whole grain.*” (Nathan, M., 13 years). Improved appeal was also suggested in the form of integrating whole grains in products that adolescents already enjoyed: “*Maybe they should make a pizza with whole grain dough, whole grain ice cream cone, or oatmeal chocolate wafers. More whole grain choco-puffs and tea biscuits too—and don’t make them the more expensive ones. They should think of more subtle and exciting ways to fit it in our everyday life!*” (Hannah, F., 15 years).

### 3.3. The SenseCam Experience

Apart from initial parental concern in the case of some participants, all participants approached were keen to take part in the research, expressing interest in SenseCam. They described it as “original”, “exciting”, and “cool” to be the first to try something new. When asked during the interviews about using SenseCam, they expressed favourable attitudes and felt this is the type of research that adolescents would engage in. They were also pleased at the notion that research was “using their language”, as a large portion of their daily life revolved around communicating with and around photos of their day: “*For us it’s all about [communicating with] pictures and uploading loads of them every day. And we just do it for fun, so it’s great to see that science is also catching up!*” (Sasha, F., 12 years). These positive appraisals were supported by observations during the interview, as the adolescents’ engagement with the picture-viewing and commenting on contextual settings was high. Participants reported they did not mind wearing the SenseCam for three days and were not concerned about privacy or unwanted attention (which few of them reported) as reporting on everyday life in photos was a norm in this age group due to the popularity of social media. These findings confirmed that SenseCam is feasible and acceptable to use in this age group to explore dietary behaviour.

## 4. Discussion

This pilot feasibility study aimed to increase understanding of young people’s whole grain consumption by using a visual, participatory method to elicit novel data and determine the feasibility and acceptability of using SenseCam technology. Findings highlighted the complex interplay of factors shaping the adolescents’ consumption, from education to family behaviour to sensory appeal. Images captured the impact of parents and online media on participants’ daily life and choices. While many poor dietary practices are explained by low motivation, this study showed that low adolescent whole grain intake may be due to the difficulty of identifying whole grain products and their health benefits as well as poor availability in and outside of the home. The participants offered creative ideas on raising awareness through online media, improved sensory appeal, increased availability and variety, and tailoring of products for young people. The findings also suggest that SenseCam was a feasible method of researching diet in young people and effective in engaging them in research; in order to capture routine but important dietary practices in everyday life, and in scaffolding a participant-led interview.

### 4.1. Adolescents, Health and Whole Grains

The adolescents were generally aware of whole grain foods, and despite a few misconceptions and issues in identification, they knew that whole grain was healthier than refined grain but were unsure why. During the interview, learning about the different varieties of whole grain and their health benefits was of significant interest to participants; learning that a certain desirable food type was in fact whole grain seemed to motivate them to try it more often in the future. Learning about different varieties of whole grain foods (other than wholemeal bread) may improve the appeal of whole grain foods and increase consumption. This is particularly important, given the centrality of taste and sensory appeal to this age group [20,21]. Although sensory appeal was ranked highly by adolescents, an appreciation of the healthiness of food did emerge [40], especially among the older participants. Their views on healthy foods were largely based on processed vs. less processed/fresh food, being preservative- and artificial colouring-free. This may be attributed to the trends being promoted online and in schools regarding preservatives and processing of foods. These findings seem to be in line with those from focus groups with adolescents [22]. It may be useful to promote whole grain to this age group by focusing on it being less processed than its refined counterpart.

### 4.2. Family as Highly Influential

When it came to food habits and nutritional information, most participants were influenced by their family members who were reported as encouraging them to improve the quality of their diet, albeit only minimally or occasionally in some cases. Pro-active or absent parental influence appeared to impact home availability of whole grain foods, knowledge, attitudes, and habitual consumption although this needs confirmation through a larger study. Participants also cited accompanying their parents to food shopping, evident in most participants’ SenseCam photos. Therefore, with the right incentives for both parents and adolescents, an active participation in shaping family (and personal) meals could be developed and directed towards an increased whole grain food availability and consumption. The conclusions drawn from these data are in line with those of existing studies on whole grain with adolescents, where habitual consumption, home availability of whole grain foods and family meal frequency were positively associated with whole grain food intake [20,21].

The participants’ statements, along with the observed patterns with whole grain consumption in different households, may contradict the common belief that peers were the most influential group for adolescents—at least when it comes to health and nutritional information [23]. Accessibility of whole grain foods at home was better than outside the home, including school. Reduced availability of whole grain and healthier food choices outside the home and at school was reported in the literature [23,40]. Participants reported a difference in whole grain consumption between weekends and weekdays, and home versus eating out. They were more likely to eat healthy at home than at school, and certainly more than eating out. This points to the need to provide a wider range of choices of whole grain foods for adolescents to purchase in school and in venues around schools.

### 4.3. School as a Starting Point for Whole Grain Promotion

In addition to increasing whole grain product availability in school canteens, schools would be a perfect setting to encourage whole grain awareness discussions and as an example to lead discussions on food processing, product normalising and low carbohydrate diets. Participants criticised the school system for lack of focus on useful well-being knowledge, a problem noted in other studies [41].

### 4.4. Teenage Culture and Importance of Social Media

Our findings suggest that peers are influential although not as much as parents; a point also highlighted in a systematic review on adolescent healthy eating interventions [23]. In our study, it was unclear why peers were not considered a major source for dietary influence, but a greater level of peer pressure was reported by younger adolescents, where desire to adhere to social norms appeared strongest. Older adolescence was marked by emergence of “interest” groups, which allowed for less pressure to conform and an increased level of autonomy and friendships based on shared values and lifestyles, including food choices [42]. These trends or differences between adolescence age-stages should be accounted for in interventions targeting younger compared with older adolescents.

Social media creates trends and priorities through celebrities sharing live images of their daily life and giving advice on YouTube and Instagram. There is a substantial focus on exercising, healthy eating, and fitness on these channels, sometimes to extremes. As teenagers are receptive to and trust their social media celebrities it would be helpful to make use of their credibility to pass a healthier whole grain message that could counteract some of the extreme diet tips and fads being promoted. Normalising or integrating whole grain promotion in an appealing way for this age group should include it being a food that would help empower their efforts in healthy weight maintenance or physical activity/sports programmes—an intervention element suggested in a systematic review on adolescents and healthy eating [23]. Moreover, efforts to promote whole grain foods based on general healthiness may be hindered by misconceptions or rumours surrounding the avoidance of all “carbohydrates” in the media, as mentioned by the participants. Acknowledging the body-image challenges facing this age group as well as the abundance of low-carbohydrate dietary advice in the media is important, and efforts to increase whole grain intake in this age group needs to address these issues.

### 4.5. SenseCam as a Valuable Tool to be Used with Adolescents

Perhaps one of the interesting findings from the use of SenseCam images during the interviews was the challenge of whole grain identification for the participants. There were instances where participants reported consuming whole grain foods, but the images would reveal otherwise. These difficulties are mainly attributed to wider challenges in the various definitions of wholegrain across the globe and reinforcing official recommendations [6,43,44]. An official definition and recommended intakes for whole grains in the UK have not yet been established nor promoted, thus such misconceptions and difficulties are to be expected [3,14]. The current study, with its use of SenseCam images, highlights the potential for this tool to explain and further understand the magnitude and complexities related to whole grain identification, as well as in the case of other food categories.

The SenseCam interviews began with participants declaring autonomy in food choice, a view which was prominent in focus groups with adolescents on whole grain intake [22]. However, the SenseCam images revealed details of daily life that shifted the conversations towards acknowledging the substantial family and home influence on food choices. They helped remind participants of instances where they had unknowingly consumed whole grains and revealed their liking of it. The images also helped remind them of details of the day, such as time spent on social media or instances of label reading, starting new interesting discussions on lifestyle and behavioural influences that may have been unlikely otherwise. SenseCam-assisted interviews therefore have the potential to overcome some of the limitations associated with traditional research methods in this age group and provide a more complete picture of barriers and enhancers. The feedback on SenseCam-assisted interviews was very positive in this age group, specifically in relation to it being a novel technology that included use of images. They also recommended using innovative technology for purposes of scientific research, to encourage adolescents to engage in research. This preference among young people to trying new technologies had been cited in previous studies [35,45], and the integration of technology in research with adolescents may allow for higher enjoyment and participation in an age group often seen as reluctant to engage in research.

### 4.6. Study Limitations

The main limitations of this study were related to the small sample of participants. Although the study is qualitative and does not claim to be representative of UK adolescents, the type of adolescent taking part in this research may not be representative of all adolescents. Furthermore, adolescents from low SES backgrounds were underrepresented. There are also limitations to the use of SenseCam. The process of obtaining ethical approval for under 18 year olds was particularly challenging, due to the multitude of privacy, confidentiality and participant inconvenience concerns [36]. There were concerns over privacy raised by some participants’ schools and family members, which had to be dealt with. This led to only three out of eight participants being able to wear the SenseCam to school and resulted in loss of information and data at school. From a practical perspective, participants also complained about the short battery life of the SenseCam which seemed to be shorter than previously reported [46] and the length of the strap which was probably designed for adults rather than children. At times this meant the camera was not at the ideal height. For this reason and also due to obstructions such as items of clothing or hair blocking the lens it is recommended that the design of SenseCam is refined for use in younger participants and clear instructions are provided to reduce the risk of poor quality images.

## 5. Conclusions

This innovative pilot study provided insight into adolescent daily life and contexts surrounding dietary choices, with particular emphasis on whole grain awareness, attitudes and consumption. Adolescents in this study were pro-active, interested and receptive to health messages and expressed the need to be targeted in ways which are relevant to their world. This could include factors related to branding, taste and texture. Participants trusted their family and their social media celebrities, and availability of whole grain foods at home was a key to increased consumption. This study established the feasibility of using SenseCam technology with young people to research their dietary practices. Young people explained their reason for participating was the chance to use an interesting visual based approach that reflected the realities of their lives. The study also shows that a one-size-fits-all approach is unlikely to be effective with adolescents, and tailored approaches for different age groups are recommended. A lack of motivation should not be assumed in this age group but an understanding of the microelements of their daily lives are necessary in order to design successful programmes to improve dietary behavior.

## Figures and Tables

**Table 1:** Descriptive details of participants using pseudonyms (*n* = 8).

Participant	Gender	Age	Ethnicity
Participant 1—Nathan	Male	13	British Asian—Indian
Participant 2—Dylan	Male	11	British White
Participant 3—Hannah	Female	15	British Black/African
Participant 4—Olivia	Female	14	British White
Participant 5—Peter	Male	13	British Asian—Chinese
Participant 6—Sasha	Female	12	British White
Participant 7—Liam	Male	16	British Mixed White background
Participant 8—Emma	Female	14	British White

## Supplementary Materials

The following are available online at https://www.mdpi.com/2072-6643/11/11/2620/s1, Table S1. Framework of concepts used as guidance and prompts during the interviews. Concepts/ideas inspired from literature on whole grain, adolescent diet, and adolescent lifestyle [21,47,48,49,50,51,52,53,54,55].

## References

[1] Slavin J., Jacobs D., Marquart L., Weimer K. (2001). The role of whole grains in disease prevention. J. Am. Diet. Assoc..

[2] Slavin J. (2003). Why whole grains are protective: Biological mechanisms. Proc. Nutr. Soc..

[3] Seal C.J., Nugent A.P., Tee E.-S., Thielecke F. (2016). Whole-grain dietary recommendations: The need for a unified global approach. Br. J. Nutr..

[4] Chanson-Rolle A., Meynier A., Aubin F., Lappi J., Poutanen K., Vinoy S., Braesco V. (2015). Systematic review and meta-analysis of human studies to support a quantitative recommendation for whole grain intake in relation to type 2 diabetes. PLoS ONE.

[5] Aune D., Keum N., Giovannucci E., Fadnes L.T., Boffetta P., Greenwood D.C., Tonstad S., Vatten L.J., Riboli E., Norat T. (2016). Whole grain consumption and risk of cardiovascular disease, cancer, and all cause and cause specific mortality: Systematic review and dose-response meta-analysis of prospective studies. BMJ.

[6] Ferruzzi M.G., Jonnalagadda S.S., Liu S., Marquart L., McKeown N., Reicks M., Riccardi G., Seal C., Slavin J., Thielecke F. (2014). Developing a standard definition of whole-grain foods for dietary recommendations: Summary report of a multidisciplinary expert roundtable discussion. Adv. Nutr. Int. Rev. J..

[7] Ye E.Q., Chacko S.A., Chou E.L., Kugizaki M., Liu S. (2012). Greater whole-grain intake is associated with lower risk of type 2 diabetes, cardiovascular disease, and weight gain. J. Nutr..

[8] Aune D., Chan D.S., Lau R., Vieira R., Greenwood D.C., Kampman E., Norat T. (2011). Dietary fibre, whole grains, and risk of colorectal cancer: Systematic review and dose-response meta-analysis of prospective studies. BMJ.

[9] Lei Q., Zheng H., Bi J., Wang X., Jiang T., Gao X., Tian F., Xu M., Wu C., Zhang L. (2016). Whole grain intake reduces pancreatic cancer risk: A meta-analysis of observational studies. Medicine.

[10] Mourouti N., Kontogianni M.D., Papavagelis C., Psaltopoulou T., Kapetanstrataki M.G., Plytzanopoulou P., Vassilakou T., Malamos N., Linos A., Panagiotakos D.B. (2016). Whole grain consumption and breast cancer: A case-control study in women. J. Am. Coll. Nutr..

[11] Jacobs D.R., Marquart L., Slavin J., Kushi L.H. (1998). Whole-grain intake and cancer: An expanded review and meta-analysis. Nutr. Cancer.

[12] Sadeghi O., Sadeghian M., Rahmani S., Maleki V., Larijani B., Esmaillzadeh A. (2019). Whole-grain consumption does not affect obesity measures: An updated systematic review and meta-analysis of randomized clinical trials. Adv. Nutr..

[13] Bjorck I., Ostman E., Kristensen M., Anson N.M., Price R.K., Haenen G.R.M.M., Havenaar R., Knudsen K.E.B., Frid A., Mykkanen H. (2012). Cereal grains for nutrition and health benefits: Overview of results from in vitro, animal and human studies in the healthgrain project. Trends Food Sci. Technol..

[14] Seal C.J., Brownlee I.A. (2015). Whole-grain foods and chronic disease: Evidence from epidemiological and intervention studies. Proc. Nutr. Soc..

[15] U.S. Department of Health and Human Services, U.S. Department of Agriculture 2015–2020 Dietary Guidelines for Americans. http://health.gov/dietaryguidelines/2015/guidelines/.

[16] Reicks M., Jonnalagadda S., Albertson A.M., Joshi N. (2014). Total dietary fiber intakes in the us population are related to whole grain consumption: Results from the national health and nutrition examination survey 2009 to 2010. Nutr. Res..

[17] Mann K., Pearce M., McKevith B., Thielecke F., Seal C. (2015). Whole grain intake in the uk remains low: Results from the national diet and nutrition survey rolling programme years 1, 2 and 3. Proc. Nutr. Soc..

[18] Mann K.D., Pearce M.S., McKevith B., Thielecke F., Seal C.J. (2015). Low whole grain intake in the uk: Results from the national diet and nutrition survey rolling programme 2008–11. Br. J. Nutr..

[19] Nelson M., Erens B., Bates B., Church S., Boshier T. (2007). Low Income Diet and Nutrition Survey: Summary of Key Findings.

[20] Pohjanheimo T., Luomala H., Tahvonen R. (2010). Finnish adolescents’ attitudes towards wholegrain bread and healthiness. J. Sci. Food Agric..

[21] Larson N.I., Neumark-Sztainer D., Story M., Burgess-Champoux T. (2010). Whole-grain intake correlates among adolescents and young adults: Findings from project eat. J. Am. Diet. Assoc..

[22] Kamar M., Evans C., Hugh-Jones S. (2016). Factors influencing adolescent whole grain intake: A theory-based qualitative study. Appetite.

[23] Shepherd J., Harden A., Rees R., Brunton G., Garcia J., Oliver S., Oakley A. (2006). Young people and healthy eating: A systematic review of research on barriers and facilitators. Health Educ. Res..

[24] Croll J.K., Neumark-Sztainer D., Story M. (2001). Healthy eating: What does it mean to adolescents?. J. Nutr. Educ..

[25] Story M., Neumark-Sztainer D., French S. (2002). Individual and environmental influences on adolescent eating behaviors. J. Am. Diet. Assoc..

[26] Pain H. (2012). A literature review to evaluate the choice and use of visual methods. Int. J. Qual. Methods.

[27] Glegg S.M. (2019). Facilitating interviews in qualitative research with visual tools: A typology. Qual. Health Res..

[28] Berry E., Kapur N., Williams L., Hodges S., Watson P., Smyth G., Srinivasan J., Smith R., Wilson B., Wood K. (2007). The use of a wearable camera, sensecam, as a pictorial diary to improve autobiographical memory in a patient with limbic encephalitis: A preliminary report. Neuropsychol. Rehab..

[29] Gemming L., Doherty A., Kelly P., Utter J., Mhurchu C.N. (2013). Feasibility of a sensecam-assisted 24-h recall to reduce under-reporting of energy intake. Eur. J. Clin. Nutr..

[30] Chen J., Marshall S.J., Wang L., Godbole S., Legge A., Doherty A., Kelly P., Oliver M., Patterson R., Foster C. (2013). Using the Sensecam as an Objective Tool for Evaluating Eating Patterns. Proceedings of the 4th International SenseCam & Pervasive Imaging Conference.

[31] Kelly P., Doherty A., Berry E., Hodges S., Batterham A.M., Foster C. (2011). Can we use digital life-log images to investigate active and sedentary travel behaviour? Results from a pilot study. Int. J. Behav. Nutr. Phys. Act..

[32] Gemming L., Rush E., Maddison R., Doherty A., Gant N., Utter J., Mhurchu C.N. (2015). Wearable cameras can reduce dietary under-reporting: Doubly labelled water validation of a camera-assisted 24 h recall. Br. J. Nutr..

[33] Kelly P., Doherty A.R., Hamilton A., Matthews A., Batterham A.M., Nelson M., Foster C., Cowburn G. (2012). Evaluating the feasibility of measuring travel to school using a wearable camera. Am. J. Prev Med..

[34] Sheats J.L., Winter S.J., Padilla-Romero P., Goldman-Rosas L., Grieco L.A., King A.C. Comparison of Passive Versus Active Photo Capture of built Environment Features by Technology Naïve Latinos Using the Sensecam and Stanford Healthy Neighborhood Discovery Tool. Proceedings of the 4th International SenseCam & Pervasive Imaging Conference.

[35] Barr M., Signal L., Jenkin G., Smith M. (2015). Capturing exposures: Using automated cameras to document environmental determinants of obesity. Health Promot. Int..

[36] Kelly P., Marshall S.J., Badland H., Kerr J., Oliver M., Doherty A.R., Foster C. (2013). An ethical framework for automated, wearable cameras in health behavior research. Am. J. Prev. Med..

[37] Nelson M., Atkinson M., Meyer J. (1997). A Photographic Atlas of Food Portion Sizes.

[38] Nelson M., Atkinson M., Meyer J. (1997). Food Portion Sizes: A User’s Guide to the Photographic Atlas.

[39] Braun V., Clarke V. (2006). Using thematic analysis in psychology. Qual. Res. Psychol..

[40] O’Neil C.E., Nicklas T.A., Zanovec M., Cho S.S., Kleinman R. (2011). Consumption of whole grains is associated with improved diet quality and nutrient intake in children and adolescents: The national health and nutrition examination survey 1999–2004. Public Health Nutr..

[41] Moon A., Mullee M., Rogers L., Thompson R., Speller V., Roderick P. (1999). Helping schools to become health-promoting environments—an evaluation of the wessex healthy schools award. Health Promot. Int..

[42] Contento I.R., Williams S.S., Michela J.L., Franklin A.B. (2006). Understanding the food choice process of adolescents in the context of family and friends. J. Adolesc. Health.

[43] Ross A.B., Kristensen M., Seal C.J., Jacques P., McKeown N.M. (2015). Recommendations for reporting whole-grain intake in observational and intervention studies. Am. J. Clin. Nutr..

[44] Mozaffarian R.S., Lee R.M., Kennedy M.A., Ludwig D.S., Mozaffarian D., Gortmaker S.L. (2013). Identifying whole grain foods: A comparison of different approaches for selecting more healthful whole grain products. Public Health Nutr..

[45] Boushey C.J., Kerr D.A., Wright J., Lutes K.D., Ebert D.S., Delp E.J. (2009). Use of technology in children’s dietary assessment. Eur. J. Clin. Nutr..

[46] Gemming L., Utter J., Mhurchu C.N. (2015). Image-assisted dietary assessment: A systematic review of the evidence. J. Acad. Nutr. Dietet..

[47] O’Dea J.A. (2003). Why do kids eat healthful food? Perceived benefits of and barriers to healthful eating and physical activity among children and adolescents. J. Am. Diet. Assoc..

[48] Bissonnette M.M., Contento I.R. (2001). Adolescents’ perspectives and food choice behaviors in terms of the environmental impacts of food production practices: Application of a psychosocial model. J. Nutr. Educ..

[49] Dennison C.M., Shepherd R. (1995). Adolescent food choice: An application of the theory of planned behaviour. J. Hum. Nutr. Diet..

[50] Krolner R., Rasmussen M., Brug J., Klepp K.-I., Wind M., Due P. (2011). Determinants of fruit and vegetable consumption among children and adolescents: A review of the literature. Part ii: Qualitative studies. Int. J. Behav. Nutr. Phys. Activ..

[51] Zeinstra G.G., Koelen M.A., Kok F.J., De Graaf C. (2007). Cognitive development and children’s perceptions of fruit and vegetables; a qualitative study. Int. J. Behav. Nutr. Phys. Activ..

[52] Kubik M.Y., Lytle L., Fulkerson J.A. (2005). Fruits, vegetables, and football: Findings from focus groups with alternative high school students regarding eating and physical activity. J. Adolesc. Health.

[53] McMackin E., Dean M., Woodside J.V., McKinley M.C. (2012). Whole grains and health: Attitudes to whole grains against a prevailing background of increased marketing and promotion. Public Health Nutr..

[54] McKinley M., Lowis C., Robson P., Wallace J., Morrissey M., Moran A., Livingstone M. (2005). It’s good to talk: Children’s views on food and nutrition. Eur. J. Clin. Nutr..

[55] Wind M., Bobelijn K., de Bourdeaudhuij I., Klepp K.-I., Brug J. (2005). A qualitative exploration of determinants of fruit and vegetable intake among 10-and 11-year-old schoolchildren in the low countries. Ann. Nutr. Metab..

