# Supplementary material for: Factors Influencing British Adolescents’ Intake of Whole Grains: A Pilot Feasibility Study Using SenseCam Assisted Interviews
Source: Nutrients. 2019 Nov 1;11(11):2620. doi: 10.3390/nu11112620 (PMC6893838; doi:10.3390/nu11112620)
Supplement: Supplementary file 1 [file nutrients-11-02620-s001.pdf]

Supplementary Materials

Table S1. Framework of concepts used as guidance and prompts during the interviews. Concepts/ideas inspired from literature on whole grain, adolescent diet, and adolescent lifestyle [21,48-56].

General Question Pointers	Probing points
Socio-demographic factors	Race/ethnicity – family socioeconomic status – living situation – economic barriers to healthy or whole grain eating
Personal factors	Knowledge/awareness – self efficacy – taste preference for whole grain – texture/mouth feel – appearance – familiarity with whole grains – perceived satiety value of whole grains – barriers to eating healthful foods – attitudes – perceived responsibility for healthful eating/body health/self-identity/body image – lifestyle – trusted sources of nutritional information (youtubers? Social media? Friends? Parents? Teachers?) – what is the first thing you look at when checking ingredient labels?
Socio-environmental factors	Support for healthful eating (from parents, friends, school, significant other) – social eating – family meal frequency – home whole grain food availability/visibility – level of control over food choices/perceptions of control – availability of whole grain in neighbourhood food outlets and varieties there – type of school attended/availability of whole grain – tv/media – access to unhealthy alternatives – sources of nutritional information
Behavioural factors	Breakfast frequency – lunch frequency – dinner frequency – fast food intake – eating on the run – food preparation/shopping – situational factors
Perceived benefits of eating whole grains (outcome expectancies): long term vs short term	Cognitive function/performance – physical sensation – psychological benefits – physical performance – increase in energy – physiological benefits: example weight – maintenance, digestive health
Major barriers and facilitators to whole grain consumption	Convenience of less healthful alternatives – internal/physiological preference – social reinforcement – reward driven/mood enhancement
Questions about SenseCam use	Enjoyment – inconveniences – practical issues encountered – reflections on experience
